# Comparative Assessment of Aspergillosis by Virtual Infection Modeling in Murine and Human Lung

**DOI:** 10.3389/fimmu.2019.00142

**Published:** 2019-02-05

**Authors:** Marco Blickensdorf, Sandra Timme, Marc Thilo Figge

**Affiliations:** ^1^Research Group Applied Systems Biology, Leibniz Institute for Natural Product Research and Infection Biology-Hans Knöll Institute, Jena, Germany; ^2^Faculty of Biological Sciences, Friedrich Schiller University of Jena, Jena, Germany

**Keywords:** virtual infection modeling, *Aspergillus fumigatus* lung infection, mouse model, human model, hybrid agent-based computer simulations

## Abstract

*Aspergillus fumigatus* is a ubiquitous opportunistic fungal pathogen that can cause severe infections in immunocompromised patients. Conidia that reach the lower respiratory tract are confronted with alveolar macrophages, which are the resident phagocytic cells, constituting the first line of defense. If not efficiently removed in time, *A. fumigatus* conidia can germinate causing severe infections associated with high mortality rates. Mice are the most extensively used model organism in research on *A. fumigatus* infections. However, in addition to structural differences in the lung physiology of mice and the human host, applied infection doses in animal experiments are typically orders of magnitude larger compared to the daily inhalation doses of humans. The influence of these factors, which must be taken into account in a quantitative comparison and knowledge transfer from mice to humans, is difficult to measure since *in vivo* live cell imaging of the infection dynamics under physiological conditions is currently not possible. In the present study, we compare *A. fumigatus* infection in mice and humans by virtual infection modeling using a hybrid agent-based model that accounts for the respective lung physiology and the impact of a wide range of infection doses on the spatial infection dynamics. Our computer simulations enable comparative quantification of *A. fumigatus* infection clearance in the two hosts to elucidate (i) the complex interplay between alveolar morphometry and the fungal burden and (ii) the dynamics of infection clearance, which for realistic fungal burdens is found to be more efficiently realized in mice compared to humans.

## Introduction

The concept of systems biology constitutes a powerful approach to investigate biological phenomena by combining wet-lab and dry-lab investigations that mutually support and complement each other (1–3). However, systems biology of infection faces problems that can interrupt the experiment-theory-cycle of systems biology (4–6). First, since *in vivo* experiments are predominantly conducted in animals, the general transferability of findings in the context of immunology to the human system is a matter of ongoing dispute (7, 8). Secondly, even in animal experiments it may be impossible to capture the spatio-temporal dynamics of infection processes. For example, in the case for lung infection *in vivo* time-lapse imaging is challenging due to animal breathing. In these cases, virtual infection modeling is of particular importance, since it has the potential to advance our knowledge despite the aforementioned limitations and to generate hypotheses that direct future experiments in a targeted manner (9, 10). In particular, building *in silico* models of infection on the available experimental data basis, gives rise to realistic to-scale models that can be used to compare the outcome of computer simulations for animal and human systems.

In this study, we use virtual infection modeling to investigate *Aspergillus fumigatus* lung infections. *A. fumigatus* is an environmentally wide-spread fungus that is an opportunistic pathogen causing severe infections in immunocompromised patients (11–14). The fungal conidia are small in size of 2–3 μm (12, 13) and can reach the alveoli in the lower respiratory tract of the lung. Because alveoli make up about 50% of the lung volume and also make the largest contribution to lung surface area, they are by far the most likely niche for infection (15). If not efficiently removed by the innate immune system, *A. fumigatus* can cause invasive pulmonary aspergillosis (IPA) with high mortality rates of 30–90% (11). The resident immune cells in the lung are alveolar macrophages (AM) that constitute the first line of immune defense by phagocytosing the inhaled conidia (11, 14, 16). Without efficient clearing by innate immunity, *A. fumigatus* conidia can undergo morphological changes: Upon contact to the surfactant layer, which covers the alveolar epithelial cells (AEC) (15), resting conidia can swell and after ~6 h start forming hyphae. These hyphae are able to penetrate the epithelial tissue of the alveolus and can thereby reach the bloodstream, from where they may disseminate and cause severe systemic infections (12, 13, 17). The first six hours after entrance of the conidia in the lung are therefore considered as a critical time frame, during which conidia need to be found in order to prevent damage of host tissue. This implies that the role of adaptive immunity can be neglected compared to a required rapid response by innate immunity, e.g., involving the complement system as well as phagocytic activity by AM and neutrophils. The condition of neutropenia, i.e., the considerable reduction in the absolute neutrophil count, poses a major risk factor for IPA (14, 18). Therefore, the nowadays increasing number of immunocompromised patients leads to a rising clinical prevalence, making *A. fumigatus* a relevant target for fungal infection research. Due to its complex interactions with the host immune system and its ability to adopt different morphologies, various levels of pathogenicity have to be considered in the development of effective therapy (13, 19).

Various mammalian species have been used for experimental research on *A. fumigatus* infection. Besides rats, rabbits, and guinea pigs, mice models have been used most extensively (20). It is important to note that—in order to provoke measurable numbers of interactions between pathogens and host cells—the experimentally applied infection doses typically are orders of magnitude higher compared to the natural inhalation dose for humans, which ranges between a few hundred and thousands of conidia per day (21–25). Thus, in addition to studying animal systems with host environments that are quite different from the human system, the significant differences in the applied infection doses need as well to be taken into consideration in the knowledge transfer from animals to humans. However, little is known about the comparability and transferability of mouse infection models in wet-lab and natural *A. fumigatus* infections in human. Therefore, in this study we compare *A. fumigatus* infection in mice and humans using virtual infection modeling to account for the respective lung morphologies and study the impact of the infection doses. In passing we note that, even though daily inhalation doses will be associated with homeostatic clearance and will typically pass unnoticed, we here use throughout the more general term infection clearance involving inflammation, tissue damage and a multifactorial host response in the case of high fungal doses.

In previous studies, we already implemented an infection model for the simulation of *A. fumigatus* infection in humans. The agent-based model (ABM) was built on an extensive experimental data basis available from literature and represents a typical human alveolus in three-dimensional continuous space (26, 27). The human alveolus was composed of AEC of type I and II, as well as of Pores of Kohn (PoK) representing connections between neighboring alveoli (28, 29). Our computer simulations revealed that AM performing random walk migration are not able to reliably detect a conidium in the alveolus before the onset of germination, i.e., before 6 h post infection (17, 26). This led to the hypothesis that a not yet experimentally identified chemotactic signal must exist that guides AM to the position of the conidium in the alveolus (26). The virtual infection model was then extended to explicitly incorporate chemokine secretion and diffusion by solving partial differential equations in a hybrid ABM (27). Scanning all unknown parameters within reasonable ranges, we determined those relevant for efficient pathogen clearance. For example, we found that a preferably high ratio of chemokine secretion by AEC with rate *s*_*AEC*_ over chemokine diffusion with diffusion coefficient *D* is required to establish a chemokine gradient that facilitates AM to detect a conidium before the onset of germination.

While these studies considered the immune response in human alveoli for daily inhalation doses of *A. fumigatus* conidia, the focus of the present study is on comparing *A. fumigatus* infections in mice and humans taking into account natural as well as experimental infection doses. Thus, we significantly adapted the agent-based virtual infection model to the to-scale morphometry of mouse alveoli. This enables generating comparative and quantitative predictions on the influence of morphological factors as well as dose-dependent effects during *A. fumigatus* infection in mice and humans.

## Results

*Aspergillus fumigatus* lung infection is commonly investigated using mouse models (20), where the pathogens can be administered in different ways (30): Intranasal deposition and intra-tracheal/intra-bronchial instillation bring the conidia directly in the nose or trachea/bronchia and are based on liquid solutions, while a more natural administration is realized in inhalation chambers with air-soluted conidia. All methods have in common that relatively high doses of 10^6^−10^8^ conidia are applied; however, the amount of conidia which is actually reaching the lower respiratory tract, i.e., the fungal burden in the alveoli of the mouse lung is found to be in the range of 10^3^−10^5^ conidia (31, 32). On the other hand, it is reported that the distribution of conidia is fairly uniform only for administration by inhalation, whereas intranasal administration is accompanied with the accumulation of conidia in specific lung sections, i.e., inducing distributions with local variations in the fungal burden (33). This implies that our *in silico* experiments need to incorporate three major issues that differ from simulations of the human infection scenario: (i) implementing the differences in the morphometry of the lung for human and mouse, (ii) scanning for a larger range of infection doses, and (iii) studying the limit of high local fungal burdens due to the non-uniform distribution of conidia for administration based on liquid solutions.

As a measure of fungal clearance, we introduced an infection score *IS*^*s* = *H,M*^, where the superscript refers to the human (*s* = *H*) or mouse (*s* = *M*) system and *IS*^*s* = *H,M*^ = 0 (*IS*^*s* = *H,M*^ = 1) implies that infections were cleared in each (none) simulations (for details see Materials and Methods section, Readout of Simulations).

### Putative Morphology-Related Impact on Infection Clearance in Humans and Mice

**Figure 1:**
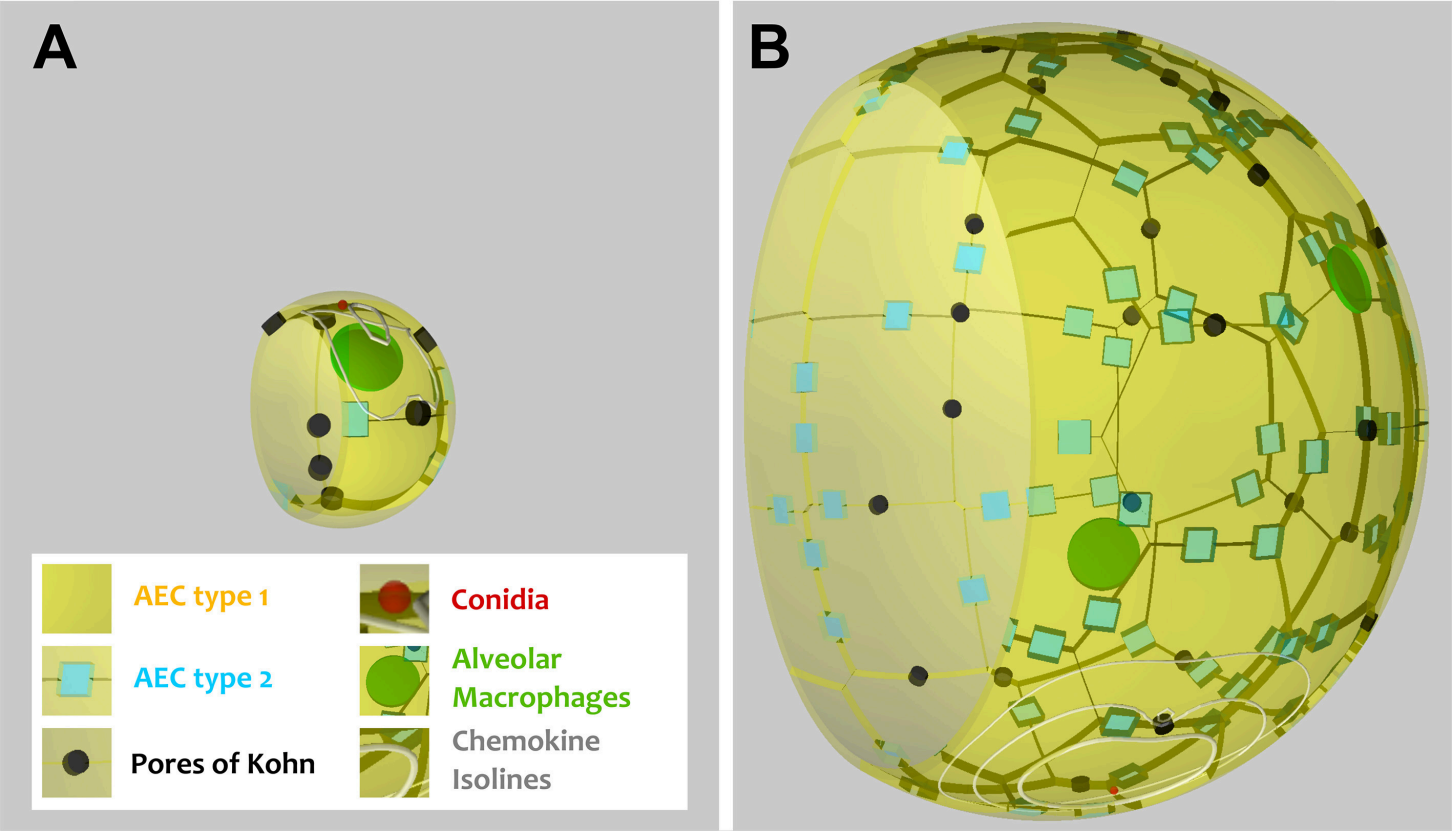
Visualization of a to-scale alveolus in the hybrid agent-based model for mouse **(A)** and human **(B)**. The alveolar entrance ring (left) and Pores of Kohn (black) represent entry/exit points for AM (green) and chemokine flow (white isolines) induced by conidium (red). Alveolar surface is covered with epithelial cells of type 1 (yellow) and 2 (blue).

As can be seen in Figure 1, the alveoli for human and mouse have been implemented as to-scale models that are composed of AEC of type I and II, as well as PoK. Given the differences in the size and composition of alveoli for the two organisms (see Table 1 and Supplementary Table 1), it can be expected that infections may be cleared with different efficiency. For example, the surface area of the human alveolus is about 20 times larger compared to that of the murine alveolus and the number of AM per alveolus is about 6 times higher in the human alveoli. This gives rise to a scanning area per AM, which is about three times higher in humans suggesting that mice could cope much better with the detection of alveolar pathogens. However, the situation is complicated by the fact the number of PoK per alveolar area is higher by a factor 5.7 in the mouse alveolus, which together with the alveolar entrance ring gives rise to an increase of the relative alveolus' open boundary length by a factor 3.4 compared to the human alveolus. On the one hand, since AM can enter and leave the alveolus only across these boundaries (28, 29), this may result in a faster infection dynamics of the murine system. On the other hand, chemotactic signaling molecules can as well flow out of the alveolus via these boundaries implying that their increased length in the murine alveolus may be of disadvantage with regard to establishing an efficient chemokine gradient. Again, this argument may only be valid for a low pathogen density in the alveolus, because for high pathogen densities the induced chemokine profile may provide an ambiguous signal for AM guidance. For the same fungal burden in mice and humans, the pathogen density is much higher in the murine alveolus, due to their much lower number and smaller size. Therefore, *A. fumigatus* may be much more efficiently cleared from the human lung. Taken together, these considerations imply that the efficiency of the infection dynamics will depend on the combination of the alveolar morphometry and the fungal burden that together impact on the chemokine profile for AM migration in a way, which is impossible to quantitatively predict without performing comparative computer simulations of to-scale models.

**Table 1:** Comparison of morphometric parameters and innate immune cells.

**Parameter**	**Human alveolus**	**Mouse alveolus (references)**
Radius of alveolus	116.5 μ*m*	26.2 ± 7.2 μ*m* (29, 34–41)
Number of type 1 AEC	48	4 (42)
Number of type 2 AEC	84	4 ± 2.4 (42–44)
Number of PoK	24	7 (45)
Type 1 AEC radius	27 μ*m*	22 μ*m*
Type 2 AEC edge length	9.34 μ*m*	8.12 μ*m* (42)
Number of alveoli per lung	4.8 × 10^8^	3.3 ± 1.3 × 10^6^ (34, 41)
Number of AM	2.1 × 10^9^	2.4 ± 0.7 × 10^6^ (42, 46)
Radius of AM	10.6 μ*m*	9.5 μ*m* (47)

*The parameters of the human alveolus were taken from the literature search by Pollmächer et al. (26) and those of the mouse alveolus have been retrieved from the indicated references*.

### Case of Low Fungal Burden: *A. fumigatus* Infection More Efficiently Cleared in Mice

**Figure 2:**
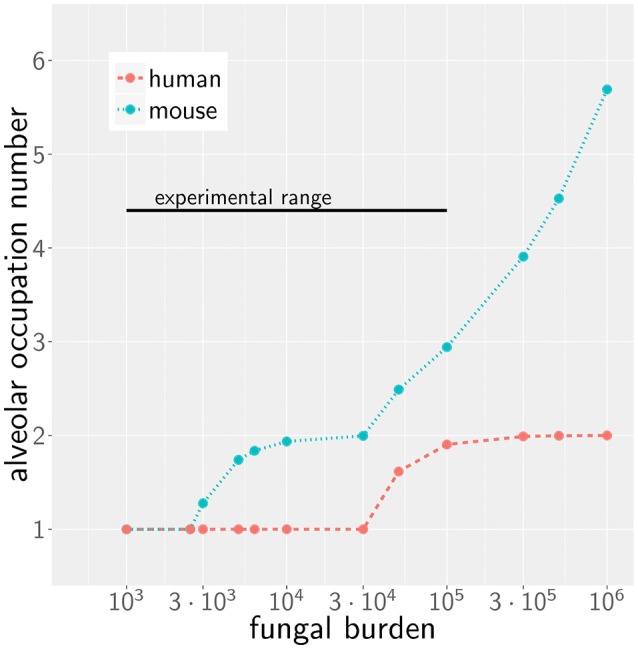
Alveolar occupation number, the maximal expected number of present conidia per alveolus, as a function of the fungal burden in mouse (blue) and human (red). Black line represents the experimental range of fungal burden, which is reached in typical mice model experiment.

We first consider the case of low fungal burden, which we define as the case where one *A. fumigatus* conidium per alveolus is the highest alveolar occupation number (AON) that is statistically expected to occur in the whole lung. The corresponding fungal burden can be derived from the binomial distribution (see Methods section for details) and is 2.5 × 10^3^ in mice and 3 × 10^4^ in humans (see Figure 2). This implies that the limit of low fungal burden covers the dose of daily inhalation for humans, but is relatively low for experimental conditions in typical mice experiments. Examples of the infection dynamics can be seen for humans and mice in Supplementary Videos 1, 2, respectively.

**Figure 3:**
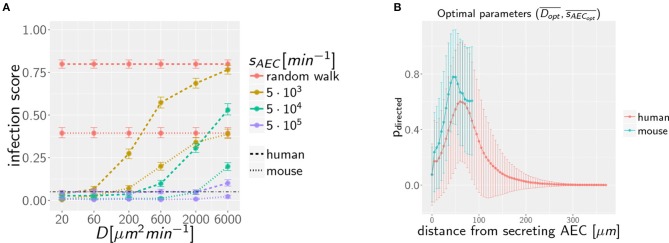
**(A)** Infection scores *IS* for random walk migration and selected examples of chemokine parameters in the limit of low fungal burden with AON = 1. Dashed-dotted black line indicates the threshold infection score at *IS*_*t*_ = 5%. Error bars represent 95%-confidence intervals received from bootstrapping. **(B)** Mean probability for directed AM migration *p*_*directed*_ following the underlying chemokine gradient as a function of the distance from the source AEC. Chemokine parameters are set to the corresponding optima $\bar{D_{opt}}$ and $\bar{s_{AEC_{opt}}}$ in mice and men.

Our previous work on *A. fumigatus* infection in human alveoli for low fungal burden revealed that a high secretion rate *s*_*AEC*_ of chemotactic molecules combined with a low diffusion coefficient *D* of the chemokine is beneficial for a small infection score *IS*^*H*^ in humans (27). In the present study, we screened the diffusion coefficient and the secretion rate in the regimes, respectively, *D* = [20, 6 × 10^3^] μ*m*^2^/min and $s_{AEC}=\left[1.5\;\times \;10^{3},\;5\times 10^{5}\right]\;min^{-1}$ for alveoli of mice and humans. The numerical results for the quantitative comparison between human and mouse is shown by the infection scores *IS*^*H,M*^ in Figure 3A. It can be observed that, for all combinations of *D* and *s*_*AEC*_, the infection score in mice is significantly smaller: *IS*^*M*^ < *IS*^*H*^. Furthermore, it can be seen that the relation of a high secretion rate and a low diffusion coefficient also leads to a more efficient infection clearance in mice. The relative difference in the infection scores of the two organisms, Δ*IS* = 1 − *IS*^*M*^/*IS*^*H*^, is in the range 50−90 %, indicating that the murine system performs always better than the human system in the limit of a low fungal burden.

### Case of Low Fungal Burden: Size of Alveolus Governs Infection Dynamics

To dissect whether the infection dynamics is governed by the chemotaxis or the alveolar size, we compared the probability of directed AM migration resulting from one conidium in the alveolus of mice and humans. The chemokine concentration itself falls off with the distance from the source AEC, i.e., the AEC in contact with the conidium. In order to avoid that AM perform mostly random walk migration, the chemokine gradient (i) must not exceed a certain value to avoid saturation of AM chemokine receptors and (ii) must not fall below a certain value to provide a detectable signal. As a qualitative measure of gradient efficiency we calculated the probability that AM follow the gradient depending on the distance to the source AEC. This probability reflects the impact of the chemokine gradient on AM migration and was computed as explained in Supplemantary Methods (see section on AM Migration) for optimal chemokine parameters $\left(\bar{D_{opt}^{s}},\;\bar{s_{AEC_{opt}}^{s}}\right)$ in the human (s = H) and mouse (s = M) system. The optimal parameters were computed from the 36 scanned parameter combinations, {*D*_1_…*D*_6_} × {*s*_*AEC*_1__…*s*_*AEC*_6__}, for the diffusion coefficient and the secretion rate as follows: Based on the simulation results in terms of the infection score *IS*_*D*_*i*_,__*s*_*AEC*_*i*___ and the limits of its respective 95%-confidence interval, we computed the optimal diffusion coefficient as $\bar{D_{opt}}=\frac{1}{\underset{i}{\sum }w_{i}}\cdot \underset{i}{\sum }w_{i}\cdot D_{i}$ with weights *w*_*i*_ = 1−*IS*_*D*_*i*_,__*s*_*AEC*_*i*___ for all those parameter combinations that had infection scores not exceeding the minimal upper limit of all confidence intervals (see Supplementary Video 3). The optimal secretion rate $\bar{s_{AEC_{opt}}}$ was determined in the same way yielding for the human host $\bar{{D_{opt}}^{H}}=34\mu m^{2}\overset{-1}{\text{ min}}$ and $\;\bar{s_{AEC_{opt}}^{H}}={1.5\times 10}^{4}{\text{min}}^{-1}$ and for the murine host $\bar{{D_{opt}}^{M}}=61\mu m^{2}\overset{-1}{\min}$ and $\bar{s_{AEC_{opt}}^{M}}={4.9\times 10}^{4}\overset{-1}{\min}$ as the optimal parameters in the limit of low fungal burden.

The probability of directed AM migration for both host systems and for their respective optimal chemokine parameters is plotted in Figure 3B. The two curves exhibit quantitative similarity suggesting that the infection dynamics in the case of a low fungal burden is mainly governed by the size of the alveolus rather than the chemokine profile itself. Thus, in contrast to the significantly larger human alveolus, AM in the murine counterpart will typically perform directed migration across the entire alveolus.

### *A. fumigatus* More Efficiently Cleared in Mice for Any Alveolar Occupation Number

**Figure 4:**
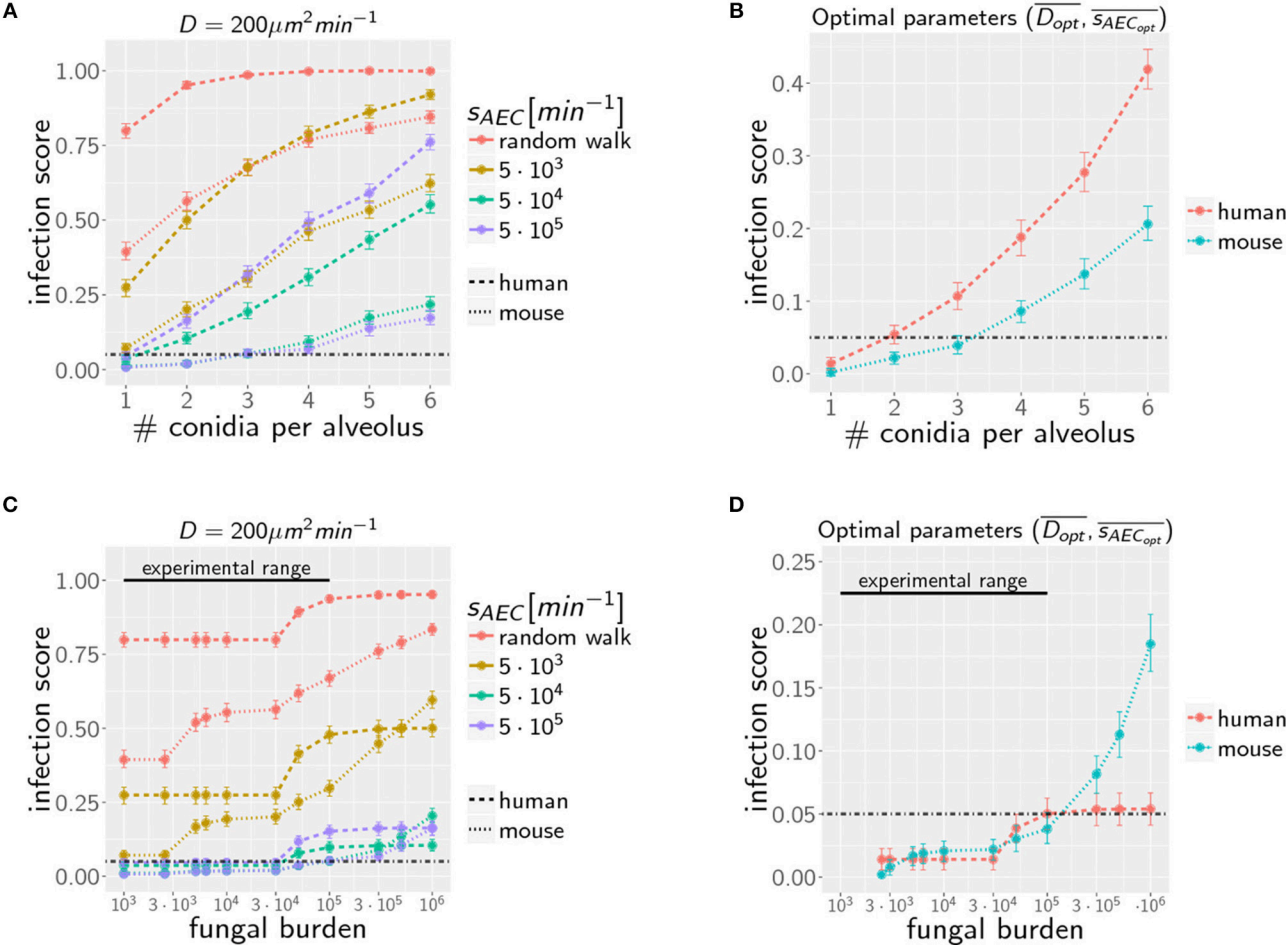
Infection scores *IS* as a function of the AON **(A,B)** and the fungal burden **(C,D)** for selected secretion parameters with diffusion coefficient $D=200\;\mu m^{2}\overset{-1}{\min}$ **(A,C)** and for optimal chemokine parameters $\bar{D_{opt}}$ and $\bar{s_{AEC_{opt}}}$ **(B,D)** in mice and men. Dashed-dotted black line indicates the threshold infection score at *IS*_*t*_ = 5%. Error bars represent 95% confidence intervals. Black line represents the experimental range of fungal burden, which is reached in typical mice model experiment.

Increasing the AON from one to higher conidia numbers, we again performed computer simulations for various infection scenarios that differ in the parameters for chemokine secretion *s*_*AEC*_ and diffusion coefficient *D*. However, multiple conidia within the alveolus can lead to more complex chemokine profiles derived from the various conidia-associated AEC that are simultaneously serving as sources of chemokine secretion. In Figure 4A the infection scores *IS* obtained from 10^3^ simulations are summarized for AON between one and six and for selected secretion rates *s*_*AEC*_, while the numerical results for the full range of studied parameter values is shown for human and mouse in Supplementary Figure 1. Parameter regimes of efficient infection clearance in these plots resemble those previously found for one conidium in the human alveolus (27), indicating that low ratios *D*/*s*_*AEC*_ are as well preferred in the mouse system.

Extending the computation of optimal chemokine parameters for one conidium to larger AON enables computing for both systems the average optimal parameter set (see Supplementary Figure 2). We obtain for one to six conidia per alveolus the averaged optimal values $\bar{{D_{opt}}^{H}}=26\pm 6.6\;\mu m^{2}min^{-1}$ and $\;\bar{s_{AEC_{opt}}^{H}}={1.1\times 10}^{4}\;\pm {6\times 10}^{3}{\;\min}^{-1}$ for the human host and $\bar{{D_{opt}}^{M}}=74\pm 22.4\;\mu m^{2}min^{-1}$ and $\bar{s_{AEC_{opt}}^{M}}={8.0\times 10}^{4}\pm {4,1\times 10}^{4}\;\overset{-1}{\min}$ for the murine host. In Figure 4B, we show that the resulting infection score *IS* as a function of the AON is always significantly lower in mice compared to humans.

### Case of High Fungal Burden: Chemokine Profile Can Deteriorate Clearance Efficiency

Due to morphometric differences between the lungs of mice and humans, the AON is not directly related to the fungal burden. This follows from our earlier statistical considerations on the highest AON that is expected to occur in the whole lung for a given fungal burden (see Figure 2) exhibiting a significant quantitative difference between mice and humans. Since the number of more than 10^8^ alveoli in the human lung exceeds that of mice by more than two orders of magnitude, even in the case of an extremely high fungal burden with 10^6^ conidia in the lung, the maximal AON for humans does not exceed two. In contrast, the same fungal burden in the lung of mice yields a maximal AON between five and six conidia in one alveolus. It thus follows that a comparison between mice and humans for the same fungal burden requires contrasting infection scenarios with different AON. Of note, our analysis focuses on the maximal AON for a given fungal burden, because it is argued that this configuration will be directly correlated with the estimated time needed to clear all occupied alveoli from the pathogen. In Figures 4C,D the numerical results for the infection score *IS* are shown for mice and humans as a function of the fungal burden, respectively, for identical chemokine parameters and for the respective optimal chemokine parameters. Supplementary Figure 3 shows the infection score *IS* as a function of the fungal burden for all the scanned parameter combinations. It can be seen by the smaller infection scores in the murine host that infections are still more efficiently cleared for the entire experimentally relevant range of 10^3^−10^5^ conidia in the lung. In Supplementary Video 3 we indicated all combinations of chemokine parameters for which the infection score reaches values below the threshold of *IS*_*t*_ = 5%.

**Figure 5:**
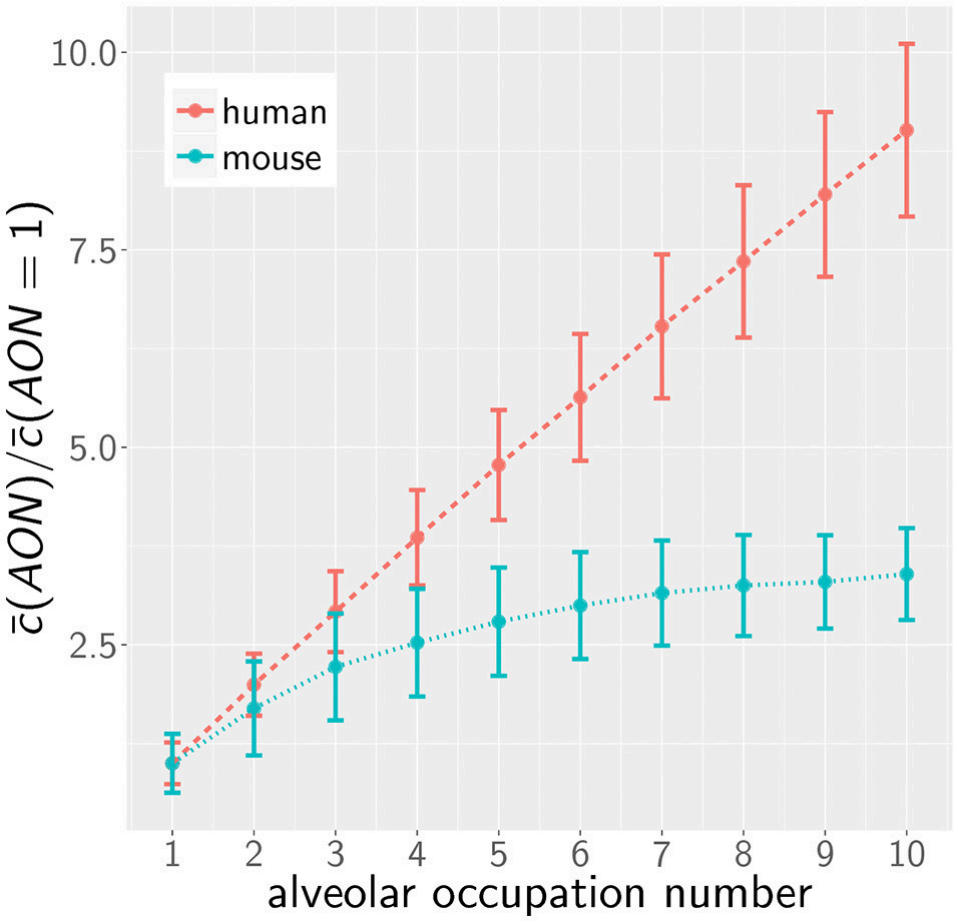
Mean of the normalized alveolar chemokine concentration as a function of the AON in mice and men obtained from simulations. Error bars represent the standard deviation of this measurement.

However, as we have mentioned before, administration of conidia based on liquid solutions is reported to be associated with higher local fungal burdens due to a more non-uniform distribution of conidia (33). It can be seen in Figure 2 for a uniform distribution of conidia that a high fungal burden in the range 10^5^−10^6^ conidia per lung is associated with an AON of two in the human system, whereas this value ranges between three and six for the murine system. Consequently, for a non-uniform distribution of conidia, such high AON can be reached in the murine lung and these can result in infection scores that are much higher than for the human system with AON of two, even if the respective optimal chemokine parameters are applied (see Figure 4B). Our spatio-temporal computer simulations of the infection scenarios reveal that higher AON are associated with chemokine profiles that deteriorate clearance efficiency. Since the mouse alveolus contains more than 10 times fewer AEC compared to the human alveolus (see Table 1), multiple randomly positioned conidia will occupy most of the alveolus' AEC associated with chemokine secretion from various source AEC. First of all, this can lead to chemokine saturation that will turn directed AM migration into random walk migration. Secondly, if the number of conidia is increased further, this will not alter the chemokine gradient anymore. Consequently, AM will perform the inefficient random walk migration until a sufficient number of conidia is detected, such that AM migration becomes again dominated by the chemokine gradient. Obviously, this complex interplay between the morphometry of the alveolus and the chemokine profile will be much less pronounced for the larger human alveolus that consists of many more AEC. To validate this hypothesis, we computed the mean values of the chemokine concentration across all alveolar surface grid points in the simulations and found that significant deviations arise between the human and mouse alveolus starting at AON of four. As can be seen in Figure 5, for AON above four the mean concentration value in the murine alveolus does change only slightly providing no additional chemotactic guidance to AM, whereas it is still increasing in the human alveolus and can provide chemotactic guidance associated with lower infection scores *IS* in the human alveolus and in the limit of fungal burdens well above the typical experimental range.

### Simulation Results Are Qualitatively Robust Against Variations of Model Parameters

In the quantitative comparison of infection scenarios in mice and humans, we so far assumed the same values for the model parameters. For example, we assumed that the chemokine secretion rates from human and murine alveolar epithelial cells are similar. However, it may be argued that this does not reflect the physiological reality correctly, since murine AEC are effectively about 33% smaller in area and may thus exhibit a reduced potential of chemokine secretion. Similarly, it is an open question today whether the postulated chemotactic signals in human and mice are transmitted by chemokines that are structural homologs and can therefore be expected to have similar diffusion coefficients in the surfactants of mice and humans. While these uncertainties cannot be avoided, we estimated the impact of variations in these parameters on the infection score in humans and mice. To this end, we calculated the relative infection score between the human and murine model Δ*IS* = 1−*IS*^*M*^/*IS*^*H*^, over all simulated parameter combinations in the experimental range of fungal burdens. Setting both diffusion parameters in humans and mice to identical values, the mouse shows lower relative infection scores with a median value of Δ*IS* = 0.49.

**Figure 6:**
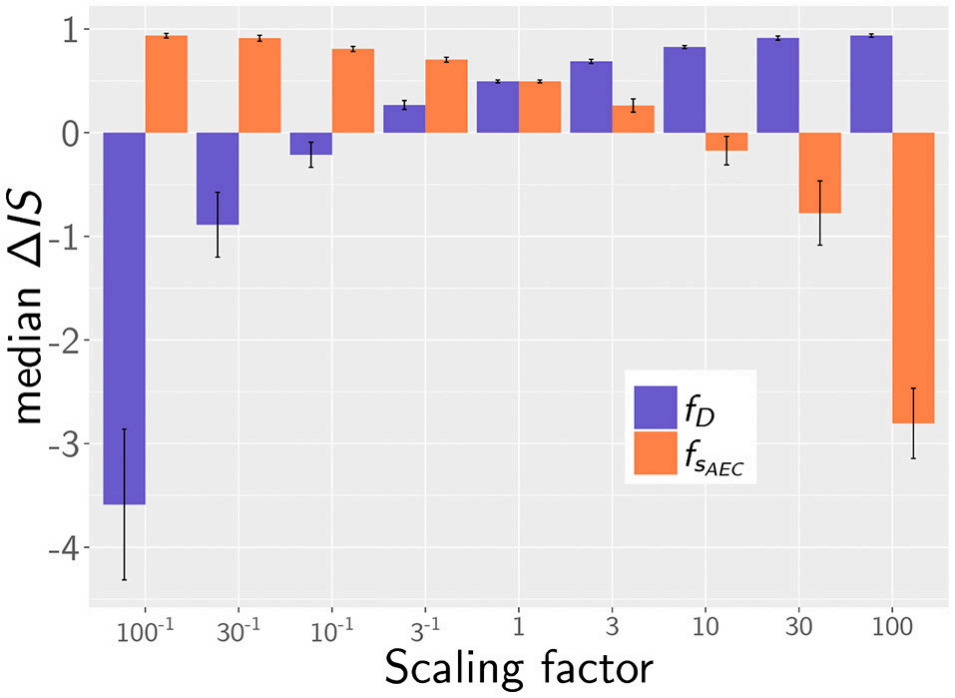
Median of the relative infection score between human and mouse, Δ*IS* = 1 − *IS*^*M*^/*IS*^*H*^, depending on the scaling factor for chemotaxis parameters: $f_{D}=\;D^{H}/D^{M}\;$ for the diffusion coefficient (purple) and $f_{s_{AEC}}=\;s_{AEC}^{H}/s_{AEC}^{M}$ for the secretion rate (orange). Error bars represent the standard errors.

Next, we analyzed the robustness of the infection outcome with regard to the diffusion coefficient and the secretion rate. To this end, we compared the infection scores for humans and mice for those simulated parameter combinations that obey the scaling factors $f_{D}=\;D^{H}/D^{M}\;$ for the diffusion coefficient and $f_{s_{AEC}}=\;s_{AEC}^{H}/s_{AEC}^{M}$ for the chemokine secretion rate. For example, comparing diffusion coefficients with scaling factor $f_{D}=3^{-1}$ (i.e., *D*^*H*^ = (20, 200, 2000) μ*m*^2^/min, *D*^*M*^ = (60, 600, 6000) μ*m*^2^/min) revealed a reduction in the median value of the relative infection score to Δ*IS* = 0.27 over the scanned fungal burdens. This indicates that the infection score in mice is higher in >50% of all selected parameter combinations, even if the diffusion coefficient is three times higher in the murine alveolus (see Figure 6). The scaling factor of the secretion rate *f*_*s*_*AEC*__ has a reversed impact on the relative infection score reflecting that a high ratio *s*_*AEC*_/*D* induces low infection scores (see Figure 6).

Taken together, our simulation results imply that our main conclusions are qualitative robust against variations in the chemotaxis parameters. As long as the associated scaling factors have values $f_{D}>10^{-1}$ or *f*_*s*_*AEC*__ < 10, the murine system still shows better infection scores in more than half of all screened fungal burdens, even if chemotactic signaling becomes deteriorated. We therefore conclude that within these limits our simulation results are qualitatively robust against variations in the chemotaxis parameters.

## Discussion

In this study, we investigated clearance of *Aspergillus fumigatus* infection from the lung of mice and humans by computer simulation of the complex interplay between alveolar morphometry and fungal burden in the dynamics of infection clearance. Since *in vivo* live cell imaging of these processes in the whole lung is still not possible today, we here extended a previously developed model of IPA in humans (25, 26) to the murine alveolus. The virtual infection model represents a realistic to-scale representation that was built on detailed experimental data available on the morphometry of the alveolus in the two hosts. Furthermore, alveolar macrophages as well as chemokine secretion and diffusion were incorporated into the model and we screened the physiologically relevant parameter ranges for as small as possible infection scores *IS*, which represent the percentage of simulations for which clearance of all *A. fumigatus* conidia from the lung took longer than 6 h.

One important finding of this study is that, for realistic fungal burdens comprising daily inhalation doses in humans as well as typical doses in mice experiments, infection clearance is more efficiently realized in mice compared to humans. This result holds true in the limit of low fungal burden, where at most one conidium is present in the alveolus, as well as for larger fungal burdens with a maximal number of two and three conidia, respectively, in the alveolus of humans and mice. As we observed before for the human system (27), a low ratio of chemokine diffusion over secretion, *D*/*s*_*AEC*_, leads to more efficient infection clearance in the murine system. However, our simulations revealed that in the limit of low fungal burden the dominating factor of efficient infection clearance in mice is the relatively short distances between AM and conidia in the relatively small murine alveolus. On the other hand, the chemokine profile played a dominant role in the limit of high fungal burden, because for four and more conidia in the relatively small murine alveolus this is associated with a featureless chemokine profile that cannot provide sufficient guidance to AM.

A quantitative comparison revealed that distinct optimal chemokine parameters exist that ensure minimal infection scores *IS* in the different alveoli of the two hosts. We therefore performed simulations comparing the infection results for both identical and optimal chemokine parameters. It should be noted that, even for the same chemotactic molecule in mice and humans, differences between optimal chemokine parameters can be induced by various factors that are different in the two hosts, such as the secretion competence of AEC and the viscosity of the alveolar surfactant. In any case, the importance of a well-established chemokine gradient as well as the functional sensing by AM is reflected by the fact that conidia, which are not detected within 6 h post infection, pose the risk of germination, invasion, and systemic infection. We also studied the case of non-uniform conidia distribution in the lung leading to locally high AON in alveoli. In this limit, which is more likely realized by the administration of conidia based on liquid solutions, our calculations predict that four and more conidia per alveolus can occur, leading to infection scores that are clearly higher in mice than in humans. However, in general, clearance of uniformly distributed conidia in the lung seems to be more efficiently realized in mice than in humans and we have demonstrated that this results are qualitatively robust over a broad range of variations in the chemokine parameters. These considerations are important with regard to the comparability and transferability of mouse infection models to the human system, e.g., with regard to estimating the efficiency of new therapeutics. Virtual infection modeling in the scope of systems biology has been applied to a broad range of biological systems and pathogens, such as bacteria (48) and fungi (9, 10, 49–53), since it provides a valuable tool to investigate infection processes that are not directly accessible in experiment. Moreover, this approach can direct future experiments by identifying key factors that govern the counterplay of infection and inflammation and require most attention. It should be mentioned that our results, indicating that AM are not able to clear the infection in the limit of a high fungal burden, are in line with previous findings based on a more phenomenological modeling approach. We applied evolutionary game theory on graphs to simulate several aspects of the immune response against *A. fumigatus* lung infection, including the complement system, phagocytosis by AM as well as recruitment and phagocytosis by neutrophils in one comprehensive model framework (54). This enabled us to reconcile the contradictory view on AM in the literature (55, 56) and predicted an infection dose-dependent switch in their function: While under low infection doses AM manage infection clearance, their role switches to a regulatory function under high infection doses by recruiting neutrophils (54).

In the future, validation of theoretical predictions needs to be addressed in experimental investigations. To date, one of the main limiting factors in understanding host response during *A. fumigatus* infections is the poor experimental accessibility and stable cultivation of alveolar tissue. However, new research approaches including organ-on-a-chip systems, which reduce the physiological complexity and bring nature closer to the simplifying virtual infection models, are promising for a better validation of e.g., alveolar epithelium properties or chemokine parameters (57–59). A lung-on-a-chip model will enable testing chemokine candidates for AM guidance, such as IL-8 that binds to the AM surface receptor CXCR2 (60). Similarly, the chemoattractant C5a is known to be activated by *A. fumigatus* conidia along the alternative pathway of the complement system (61, 62) and is able to trigger the secretion of macrophage inflammatory protein-2 and neutrophil chemoattractant-1 by AEC (63). Once chemokine parameters will have been identified and inflammatory conditions in terms of cytokine profiles will be accessible, the next step will be to extend the hybrid ABM toward neutrophil recruitment and an explicit phagocytosis model along the lines of our previous investigations based on evolutionary game theory (54). This will allow for the investigation of migration and phagocytic dynamics of AM, neutrophils and AEC in the alveolar environment during the interaction with pathogens. Furthermore, morphological changes of *A. fumigatus* including swelling and hyphae formation have a strong impact on phagocytosis of the fungus (17, 64) and can be included in such a virtual infection model. A further advancement will be in the scale-up of the alveolus to the higher organizational units of alveolar sacs for a more comprehensive simulation of infection scenarios.

## Materials and Methods

In this study, we extended our previously developed ABM of *in silico* infections by *Aspergillus fumigatus* in the human alveolus (26, 27) to the mouse alveolus in order to perform comparative analyses. The ABM is a spatio-temporal multi-scale model that simulates host-pathogen interactions on the cellular and molecular level. Thus, cells like the fungal conidia and AM are simulated as individual agents that migrate and interact in a rule-based fashion, while the chemokine secretion by AEC and the molecular diffusion of chemokines is simulated using partial differential equations. Chemokines are uniformly secreted with rate *s*_*AEC*_ at the surface of each AEC, which is associated with at least one conidium. The implementation of the ABM is described in more detail in the Supplementary Material, while here the focus is on the main aspects associated with the extension to the mouse alveolus.

### Morphometry of the Mouse Alveolus and Implementation

A comprehensive literature research was performed to design the virtual infection model of the mouse alveolus as realistic as possible. The most important morphology parameters are summarized and compared with the human alveolus in Table 1, from which other characteristics can be derived (see for examples Supplementary Table 1). For example, it can be seen that the radius (surface area) of a typical human alveolus is about 4.5 (20)-fold larger compared to a murine alveolus. The numbers of AEC of type 1 and 2 differ significantly in both organisms, i.e., a human alveolus contains about 12.0-fold more type 1 and 21-fold more type 2 AEC. Furthermore, the number of PoK is about 3.4 times higher in the human alveolus. A video of both model alveoli is provided in the (Supplementary Videos 1, 2).

### Implementation of Mouse Alveolus in Virtual Infection Model

The ABM was adjusted for the implementation of the mouse alveolus with parameters as summarized in Table 1 and Supplementary Table 1. This also required changes in the algorithm for cell positioning on the alveolar surface. Type 1 AEC were placed as described before around the three-quarter sphere (see Supplementary Material for details). Previously, type 2 AEC and PoK were placed at the borders between type 1 AEC. However, due to the larger ratio of type 2 AEC and PoK with respect to type 1 AEC in mice, the positioning of PoK and type 2 AEC had to be changed. We adjusted the position of type 2 AEC and PoK uniformly across the whole border of the type 1 AEC. While these changes in the cell positioning were required for realistic configurations of mouse alveolus morphometries, quantitative results of the ABM for the human alveolus remained within the 95%-confidence interval. Moreover, the smaller size of the mouse compared to the human alveolus required adjustment of the Delaunay-triangulated grid, on which the diffusion equation is solved (27). The number of grid points could be reduced from 10^4^ in the human alveolus to only 5.1 × 10^2^, keeping the spatial resolution in the mouse alveolus the same as in the human system (see Supplementary Table 1).

### Readout of the Simulations

As a measure of fungal clearance we compute for various infection scenarios the first-passage-time (FPT) of AM, i.e., the time required for migrating AM to find all conidia in a particular alveolus (26, 27). The relation between the FPT and the time point of conidia germination, which corresponds to about 6 h post conidia arrival, is obtained from repeating the simulation of each infection scenario 10^3^ times. From the corresponding FPT distribution, we then compute an infection score, *IS*, as the percentage *p* of simulations with FPT above 6 h: *IS*^*s* = *H,M*^ = *p*(*FPT* > 6 *h*), where the superscript refers to the human (*s* = *H*) or mouse (*s* = *M*) system and *IS*^*s* = *H,M*^ = 0 (*IS*^*s* = *H,M*^ = 1) implies that conidia were cleared in each (none) of the 10^3^ simulations. The various infection scenarios correspond to scanning the parameter space in terms of AM migration, chemokine secretion, and diffusion, as well as conidia infection doses in alveoli of mice and humans.

### Comparison of Fungal Burden

For a given fungal burden δ, the conidia are distributed across all alveoli *n*_*alv*_ of the host's lung. Assuming an independent and uniform distribution of these conidia, we can describe the probability of having *n*_*con*_ conidia present in one alveolus by the Binomial distribution *B*_*con*_(δ, *p, n*_*con*_) with probability of $p=\frac{1}{n_{alv\;}}$ for δ repeats. To estimate the maximal AON that is associated with a specific fungal burden, we computed *n*_*con*_ from the $1-\frac{1}{n_{alv}}$-quantile of the distribution *B*_*con*_(δ, *p, n*_*con*_). The resulting number corresponds to the maximal AON that can be expected to occur in the whole lung for a specific fungal burden (see Figure 2). The corresponding *IS* was determined by linear interpolation of the results from our simulations for various AON.

## Data Availability Statement

The raw data supporting the conclusions of this manuscript will be made available by the authors, without undue reservation, to any qualified researcher.

## Author Contributions

MTF conceived and designed this study. MTF provided computational resources. Data processing, implementation and application of the computational algorithm were done by MB and ST. MB, ST, and MTF evaluated and analyzed the results of this study. MB, ST, and MTF drafted the manuscript and revised it critically for important intellectual content and final approval of the version to be published. MB, ST, and MTF agree to be accountable for all aspects of the work in ensuring that questions related to the accuracy or integrity of any part of the work are appropriately investigated and resolved.

### Conflict of Interest Statement

The authors declare that the research was conducted in the absence of any commercial or financial relationships that could be construed as a potential conflict of interest.
