# Supplementary material for: Comparative Assessment of Aspergillosis by Virtual Infection Modeling in Murine and Human Lung
Source: Front Immunol. 2019 Feb 5;10:142. doi: 10.3389/fimmu.2019.00142 (PMC6370618; doi:10.3389/fimmu.2019.00142)
Supplement: Supplementary file 4 [file Data_Sheet_1.PDF]

Supplementary Material

Comparative assessment of aspergillosis by virtual infection modeling in murine and human lung

Marco Blickensdorf, Sandra Timme and Marc Thilo Figge *

*** Correspondence:** Corresponding Author: thilo.figge@leibniz-hki.de

1 Supplementary Methods

In this study, we implemented a to-scale model of the murine alveolus following our previous work on the implementation of the human alveolus. For details on the hybrid agent-based model (ABM), the reader is referred to the extended description of this model in the work by Pollmächer *et al.*(Pollmächer and Figge, 2014, 2015). This Supplementary Information presents essential aspects of the algorithmic implementation that are important in view of the current study.

1.1 Alveolus Setup

In close analogy to our previous work(Pollmächer and Figge, 2014, 2015), the human and murine alveoli were modeled as three-dimensional three-quarter spheres, with radius $r_{Alv}^{H} = 116.5\ \mu m$ and $r_{Alv}^{M} = 26.2\ \mu m$, respectively. The polyhedral shape of the alveolus was approximated by a spherical representation with surface points $\vec{x} = c(r_{Alv}, \vartheta, \varphi)$ and an entrance ring at the lower threshold value of the polar angle $\vartheta_c \leq \vartheta \leq \pi$. Alveolar epithelial cells (AEC) of realistic dimensions were placed at the inner surface of the alveolus as follows: Centroids of type 1 AEC were placed in an equidistant fashion using a Voronoi tessellation to project cells on the curved geometry of the alveolus. Type 2 AEC were placed randomly on the border between two neighboring type 1 AEC. The same procedure was applied for the positioning of Pores of Kohn. All cells were represented with realistic cell sizes and in realistic amounts as obtained from an in-depth literature search (see Table 1 and Supplementary Table S1).

1.2 Chemokine Secretion and Diffusion

The hybrid ABM(Pollmächer and Figge, 2014, 2015) was used to simulate chemokine secretion and diffusion on an equidistant grid of points on the surface of the alveolus. Placing n points uniformly on a spherical surface is known as the Thompson problem(Thomson, 1904) and we used established algorithms(MacWilliam and Cecka, 2013) to generate a near-equidistant solution of a grid with 10000 points and 513 points, respectively, for the human and murine alveolus, *i.e.* keeping the same spatial resolution in both systems. Chemokine diffusion was modeled solving the reaction-diffusion equation on the grid. The reaction-diffusion equation

$$\frac{\delta c(\vec{r},t)}{\delta t} = D\Delta c(\vec{r},t) - \lambda c(\vec{r},t) + S(\vec{r},t) - Q(\vec{r},t)$$

describes molecule diffusion with diffusion coefficient D and molecule degradation with degradation rate λ of the concentration $c(\vec{r},t)$ at position $\vec{r}$ and time point t. Furthermore, the terms $S(\vec{r},t)$ and $Q(\vec{r},t)$ describe chemokine secretion and consumption at position $\vec{r}$ and time point t, where molecule consumption involves molecule-receptor binding by alveolar macrophages. Chemokine secretion was induced at all grid points on the surface of the AEC associated with a conidium. The reaction-diffusion equation was numerically integrated in time using a finite difference method for unstructured grids, as described by Sukumar(Sukumar, 2003). When multiple conidia were present in the alveolus, all associated cells secrete chemokines with the same secretion rate. When a conidium was found by an alveolar macrophage (AM) the associated cell stopped chemokine secretion.

1.3 AM Migration

Migration of AM was realized along surface vectors of length $v\Delta t$, where v denotes the macrophage speed and Δt refers to the time step of the simulation. A new migration direction was chosen after persistence time t_p, either randomly to simulate random walk migration or biased by chemotactic signals to simulate directed AM migration. AM sensing of the chemokine concentration was realized by the receptor-ligand model that was previously introduced by Guo and Tay (2008)(Guo et al., 2008) and Guo *et al.*(Guo and Tay, 2008) and applied here to the spherical alveolar surface. This model consisted of an independent system of three differential equations reflecting the state of ligands and receptors on the AM surface. In short, the chemokine receptors were able to bind free chemokines with binding rate k_b, building a receptor-ligand complex, which was internalized by the AM with rate k_i and finally recycled and re-expressed on the cellular surface with rate k_r. The most favorable direction of migrating AM was determined by computing the sum of weighted gradients over one period of directional persistence. Then, after each period of directional persistence, the respective AM migrates in the direction of the weighted cumulative gradient $\vec{g}_{AM}$ with probability $p_{directed} = \min(||\vec{g}_{AM}||\sigma_{AM}, 1)$, where the sensitivity factor σ_{AM} was determined by Tranquillo *et al.*(Tranquillo et al., 1988). For all simulations we assumed that AM migrate at speed of $v = 4\frac{\mu m}{min}$ and with persistence time $t_p = 1\ min$.

1.4 Agent Distribution

AM were placed randomly on the inner surface of the alveolus. Placing n_{AM} AM uniformly over n_{Alv} alveoli leads to the binomial distribution $B_{AM}(n_{AM}, p, k) = \binom{n_{AM}}{k} p^k (1-p)^{n_{AM}-k}$, where the probability $p = 1/n_{alv}$ refers to the probability to have k AM per alveolus. In close analogy, the probability to have j conidia present in one alveolus is $B_{con}(n_{con}, p, j)$. The alveolar system was implemented with absorbing boundaries, *i.e.* each agent that crosses a system boundary was taken out of the system. To account for these randomly exiting AM and to preserve the binomial distribution $B_{AM}(n_{AM}, p, k)$, new AM were inserted into the system with exponentially distributed waiting time $t_{wait} = \frac{1}{\lambda_{in}} \ln(\frac{1}{u})$, where u is uniformly distributed in $(0,1]$. The input rate λ_{in} is calibrated for each set of migration parameters (v and t_p) and alveolar system (murine and human). The entry point of newly entering macrophages was modeled to depend on gradient of the local chemokine concentration, $||\vec{g}^b_{AM,i}||$, with probability $p_i = \frac{||\vec{g}^b_{AM,i}||}{||\vec{g}^b_{AM,max}||}$ at entry point i and $||\vec{g}^b_{AM,max}||$ is the maximum gradient of all boundary points. The acceptance-rejection method is applied in the following way: An entry point is accepted if $p_i < l$ with a randomly chosen l in [0,1] from a uniform

distribution for each candidate position; otherwise a new position is chosen randomly over all boundary points of the alveolus as the next candidate entry point.

1.5 Implementation

The simulation framework was implemented in $C++$ to provide maximum expandability. Model dynamics as agent migration, interaction handling and diffusion were realized by asynchronous random-order updating for each time step independently. Two agents interact if their associated spheres were overlapping. In case of contact between an AM and a conidium, the conidium was assumed to be phagocytosed by the AM.

1.6 Simulation Result Robustness

We provide a statistical estimate on how reliable our results are. As explained before, we run each simulation for a particular set of model parameters 10^3 times. One option to estimate the robustness of the resulting readouts would be to repeat these 10^3 simulations multiple times and to calculate statistical measures. Since this would be very time consuming, we decided to bootstrap(Efron and Tibshirani, 1986) the simulation results as follows: We randomly chose a set of 10^3 simulations out of the 10^3 computed simulations with replacement and computed readouts like the infection score IS for a number B of repetitions. From the resulting IS distribution we calculated the 95%-confidence interval as an estimation of the real quantiles. We here chose $B = 300$ to produce stable results.

To evaluate the results of the bootstrapping procedure, we compared it to an estimated standard deviation, which we received from 10 repetitions of 10^3 simulations for the human and mouse system with 1 conidium for all parameter combinations of chemotactic signaling. The standard deviation in these 72 simulations differed on average by 6% between the bootstrapped and the repetition estimates, indicating that our bootstrapping procedure produced valid results.

1.7 References

Efron, B., and Tibshirani, R. (1986). Bootstrap Methods for Standard Errors, Confidence Intervals, and Other Measures of Statistical Accuracy. *Stat. Sci.* 1, 54–75. doi:10.1214/ss/1177013815.

Guo, Z., Sloot, P. M. A., and Tay, J. C. (2008). A hybrid agent-based approach for modeling microbiological systems. *J. Theor. Biol.* 255, 163–75. doi:10.1016/j.jtbi.2008.08.008.

Guo, Z., and Tay, J. C. (2008). *Granularity and the Validation of Agent-based Models*. San Diego, CA: Simulation Councils, Inc doi:10.1145/1400549.1400568.

MacWilliam, T., and Cecka, C. (2013). CrowdCL: Web-based volunteer computing with WebCL. in *2013 IEEE High Performance Extreme Computing Conference (HPEC)* (IEEE), 1–6. doi:10.1109/HPEC.2013.6670348.

Pollmächer, J., and Figge, M. T. (2014). Agent-based model of human alveoli predicts chemotactic signaling by epithelial cells during early Aspergillus fumigatus infection. *PLoS One* 9, e111630. doi:10.1371/journal.pone.0111630.

Pollmächer, J., and Figge, M. T. (2015). Deciphering chemokine properties by a hybrid agent-based model of Aspergillus fumigatus infection in human alveoli. *Front. Microbiol.* 6, 503. doi:10.3389/fmicb.2015.00503.

Sukumar, N. (2003). Voronoi cell finite difference method for the diffusion operator on arbitrary unstructured grids. *Int. J. Numer. Methods Eng.* 57, 1–34. doi:10.1002/nme.664.

Thomson, J. J. (1904). XXIV. *On the structure of the atom: an investigation of the stability and periods of oscillation of a number of corpuscles arranged at equal intervals around the circumference of a circle; with application of the results to the theory of atomic structure. London, Edinburgh, Dublin Philos. Mag. J. Sci.* 7, 237–265. doi:10.1080/14786440409463107.

Tranquillo, R. T., Lauffenburger, D. A., and Zigmond, S. H. (1988). A stochastic model for leukocyte random motility and chemotaxis based on receptor binding fluctuations. *J. Cell Biol.* doi:10.1083/jcb.106.2.303.

2 Supplementary Videos

2.1 Video S1 – Human Alveolus Model Video

The video shows one exemplary simulation in the to-scale hybrid ABM for the human alveolus. The video starts with a rotation of the alveolus. The simulation of an infection scenario with one conidium starts when the time stamp is visible. AM (green) migrating towards the conidium (red) along the chemokine gradient (white isolines) produced by the source AEC. The conidium is found by an AM after 55 minutes in this simulation.

2.2 Video S2 – Mouse Alveolus Model Video

The video shows one exemplary simulation in the to-scale hybrid ABM for the murine alveolus. The video starts with a rotation of the alveolus. The simulation of an infection scenario with one conidium starts when the time stamp is visible. AM (green) migrating towards the conidium (red) along the chemokine gradient (white isolines) produced by the source AEC. The conidium is found by an AM after 21 minutes in this simulation.

2.3 Video S3 – Infection scores and parameter optima

The video provides a slide show of simulation results for increasing alveolar occupation number comparing the human (left) and mouse (right) system. The infection score is represented in a color-coded fashion as a function of all chemokine parameter combinations. Small grey dots represent all those data points that did not exceed the minimal upper limit of all 95%-confidence intervals. Gold points indicate their respective weighted mean. A black border around a data point indicates an infection score below the threshold $IS_t = 5\%$.

3 Supplementary Figures

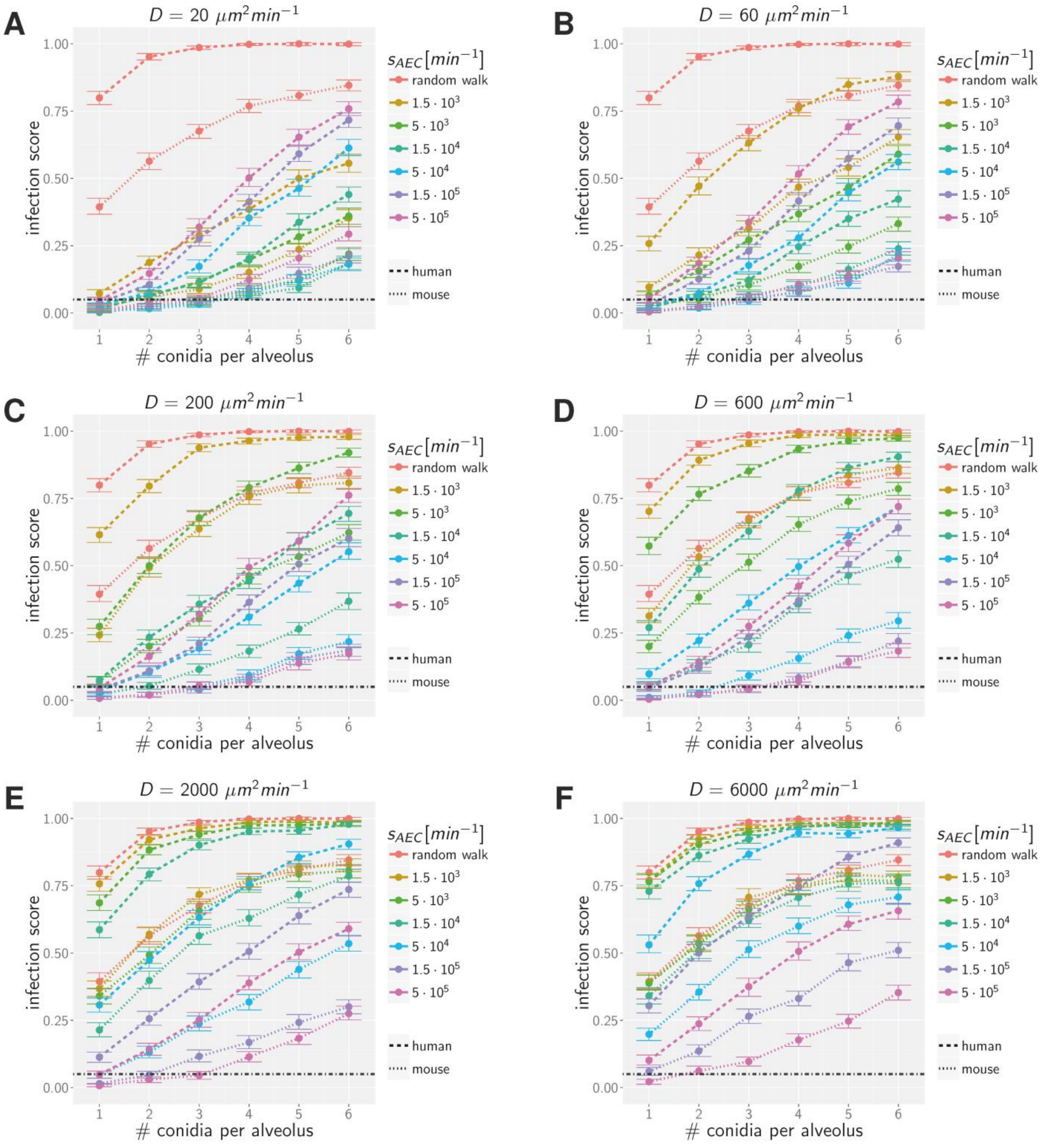

Supplementary Fig. S1: Infection scores for all scanned alveolar occupation numbers and all combinations of chemokine parameters in the human (dashed line) and mouse (dotted line) alveolus. Black dashed-dotted line indicates an infection score of $IS_t = 5\%$. Error bars represent the 95%-confidence interval.

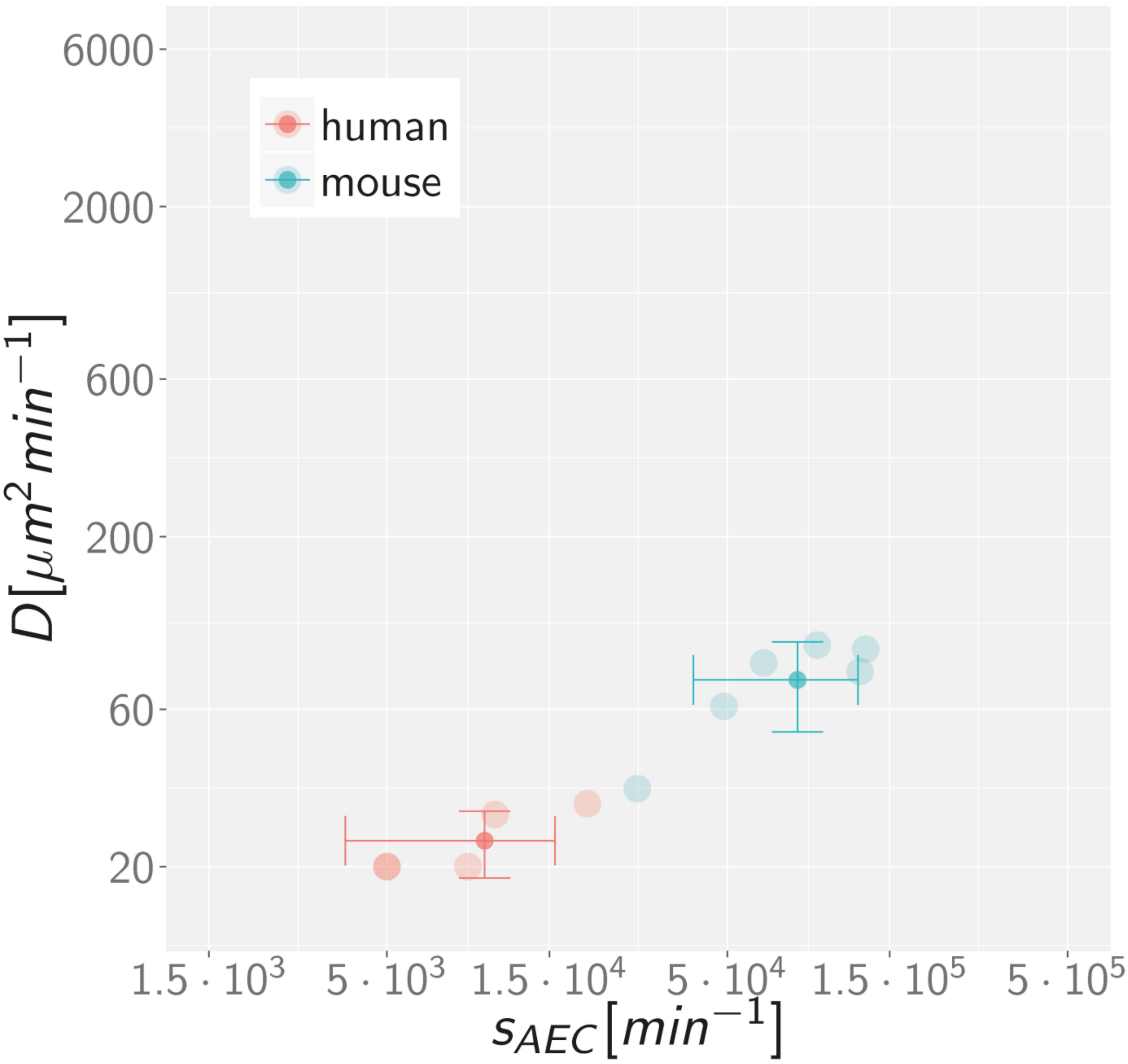

Supplementary Fig. S2: Optimal chemokine parameters for alveolar occupation numbers AON = [1,6] in the human and mouse system. For the respective mean with standard deviations across the six data points, we the values $\overline{D_{opt}}^{H} = 26 \pm 6.6\ \mu m^2 min^{-1}$ and $\overline{s^{H}_{AEC_{opt}}} = 1.1 \times 10^4 \pm 6 \times 10^3\ min^{-1}$ for the human host and $\overline{D_{opt}}^{M} = 74 \pm 22.4\ \mu m^2 min^{-1}$ and $\overline{s^{M}_{AEC_{opt}}} = 8.0 \times 10^4 \pm 4{,}1 \times 10^4\ min^{-1}$ for the murine host.

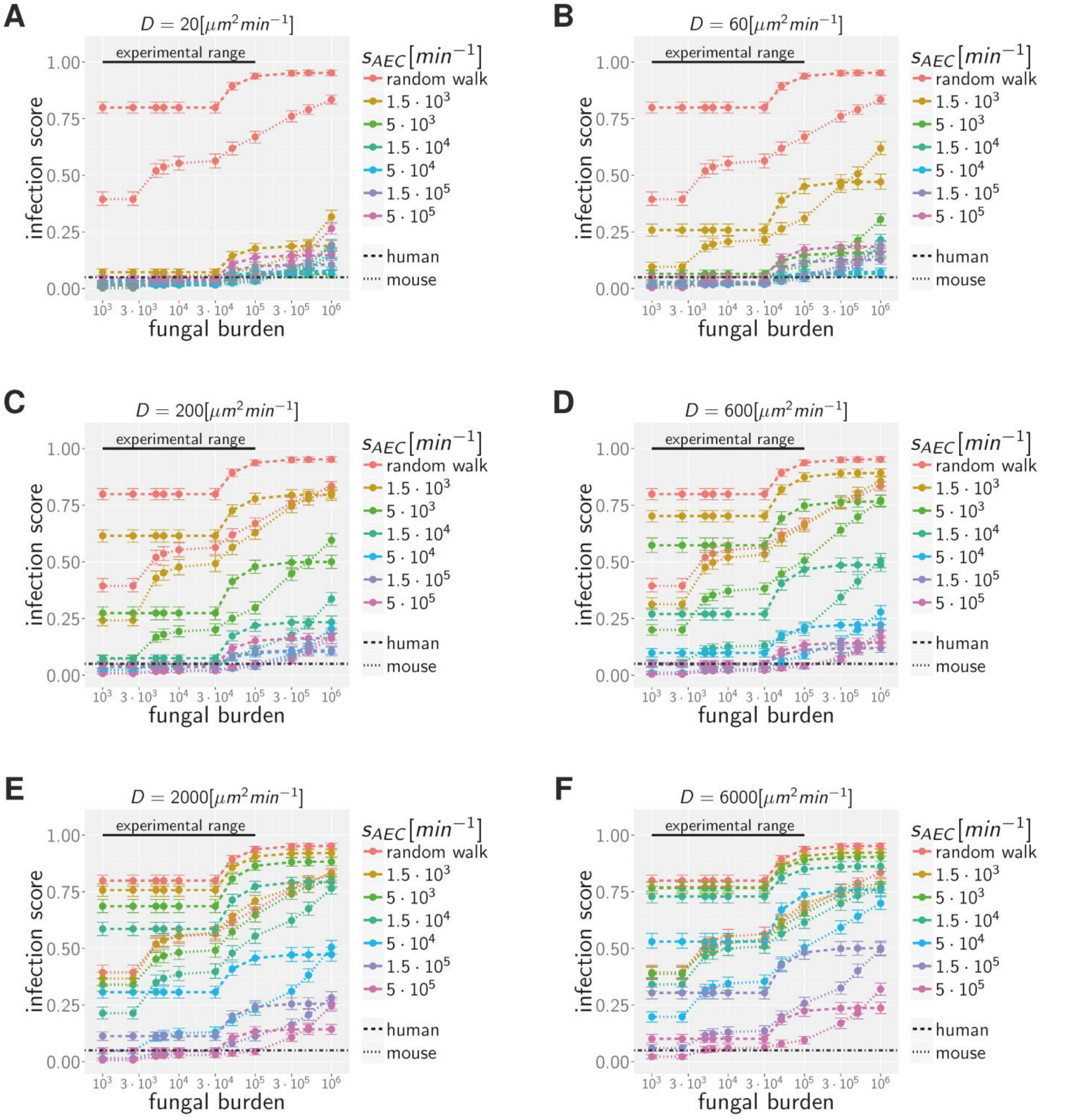

Supplementary Fig. S3: Infection scores for all scanned fungal burdens and all combinations of chemokine parameters in the human (dashed line) and mouse (dotted line) alveolus. Black dashed-dotted line indicates an infection score of $IS_t = 5\%$. Error bars represent the 95%-confidence interval.

4 Supplementary Tables

Supplementary Table 1: Average characteristics derived per alveolus for the human and murine system.

Parameter	Human	Mouse	Mouse/Human [%]
Surface area	$127 \times 10^3\ \mu m^2$	$6.5 \times 10^3\ \mu m^2$	5.1
Volume	$5\ 588 \times 10^3\ \mu m^3$	$64 \times 10^3\ \mu m^3$	1.1
Length of boundary	$743.2\ \mu m$	$128.2\ \mu m$	17.2
Surface to boundary ratio	$170.1\ \mu m$	$50.7\ \mu m$	29.8
Mean AM number	4.38	0.74	16.9
Surface area per AM	$28.9 \times 10^3\ \mu m^2$	$8.8 \times 10^3\ \mu m^2$	30.4
Mean distance to boundary*	$95.4\ \mu m$	$21.3\ \mu m$	22.3
Surface grid points	10 000	513	5.13

*The mean distance to boundary was computed for random points in the respective alveolar systems measuring their distance to the closest border point from 1000 random realizations.
